# Glucose Metabolism and Cognitive Decline in Progressive Supranuclear Palsy and Corticobasal Syndrome: A Preliminary Study

**DOI:** 10.3390/jcm13020465

**Published:** 2024-01-14

**Authors:** Natalia Madetko-Alster, Dagmara Otto-Ślusarczyk, Marta Struga, Michał Kutyłowski, Agnieszka Drzewińska, Karolina Duszyńska-Wąs, Bartosz Migda, Piotr Alster

**Affiliations:** 1 Department of Neurology, Medical University of Warsaw, Kondratowicza 8, 03-242 Warsaw, Poland; piotr.alster@wum.edu.pl; 2 Department of Biochemistry, Medical University of Warsaw, Banacha 1, 02-097 Warsaw, Poland; dagmara.otto@wum.edu.pl (D.O.-Ś.); marta.struga@wum.edu.pl (M.S.); 3 Department of Radiology, Mazovian Brodno Hospital, Kondratowicza 8, 03-242 Warsaw, Poland; michael.kutylowski@gmail.com; 4 Department of Neurology, Mazovian Brodno Hospital, Kondratowicza 8, 03-242 Warsaw, Poland; agnieszka.drzewinska@gmail.com (A.D.); karolina.duszynska@gmail.com (K.D.-W.); 5 Diagnostic Ultrasound Lab, Department of Pediatric Radiology, Medical University of Warsaw, 02-091 Warsaw, Poland; bartosz.migda@wum.edu.pl

**Keywords:** atypical Parkinsonian syndrome, progressive supranuclear palsy, corticobasal syndrome, cognitive impairment, impaired glucose metabolism, glycemic variability

## Abstract

Multiple studies have analyzed the possible correlations between diabetes and Alzheimer’s disease. Less is known about the context of cognitive deterioration among patients with atypical Parkinsonian syndromes and glucose metabolism impairment. The aim of this study was to evaluate the association between the impaired glucose metabolism and cognitive decline among patients with progressive supranuclear palsy (PSP) and corticobasal syndrome (CBS). The study included 22 patients with PSP and CBS with disease durations varying from 3 to 6 years. The levels of glycated hemoglobin (HbA1C), fasting blood glucose, fasting C-peptide and the presence of microalbuminuria were evaluated, and oral glucose tolerance tests (OGTT) were performed. Based on the OGTT results, the glycemic variability, mean glycemia, glycemia standard deviation (SD) and coefficient of variation (%CV) were calculated. All patients underwent a three-Tesla brain magnetic resonance (MRI) examination and neuropsychological cognitive assessment with the use of standardized scales: Montreal Cognitive Assessment (MoCA), Mini-Mental State Examination (MMSE) and Frontal Assessment Battery (FAB). A statistical analysis revealed that poor control of glycemia with high glycemic variability and increased atrophy of the medial temporal lobe among patients with PSP and CBS correlated with worse cognitive performance independent of age or sex, even among patients who did not fulfill the criteria for diabetes. The study results indicate the importance of glucose metabolism control and optimal treatment in the context of cognition maintenance among patients with PSP and CBS. Due to the relatively small number of analyzed patients, the issue requires further assessment. To the best of our knowledge, this is the first study discussing the role of glycemic variability in atypical Parkinsonian syndromes.

## 1. Introduction

Progressive supranuclear palsy (PSP) and corticobasal syndrome (CBS) are considered as atypical Parkinsonian syndromes. In the case of PSP, it is neuropathologically defined as a four-repeat tauopathy (4R tau) [1]; however, CBS is a clinical entity with multiple possible pathologies. CBS is often associated with corticobasal degeneration (CBD), which is a neuropathological diagnosis based on indicating the presence of hyperphosphorylated 4R tau deposits. PSP is currently defined as a group of clinical entities differing in the context of the presence and intensity of certain diagnostic features. PSP-Richardson’s syndrome (PSP-RS) is the most common subtype of the disease and is defined by early deterioration in oculomotor functions, postural instability and akinesia. The second most common subtype—PSP—Parkinsonism Predominant (PSP-P) has a more beneficial course and is linked with a levodopa response in its primary stage [1]. Other subtypes, though indicated in the criteria of diagnosis, are very rare and described briefly in the literature. CBS is a heterogeneous group of diseases based on numerous pathologies, among which CBD is associated with less than 50% of cases. The clinical differentiation of subtypes of PSP and CBS is often affected by obstacles related to overlapping manifestations. Moreover, the use of a supplementary examination does not provide sufficient in vivo data enabling a definite in vivo diagnosis.

Cognitive dysfunction is one of four functional domains defined in PSP diagnostic criteria [1]. It is also described as common among CBS patients as it reflects higher cortical features’ impairments characteristic for this entity [2]. Among other symptoms of PSP and CBS, cognitive decline is one of the most disabling and responsible for reducing patients’ quality of life as well as increasing depression rates, the burden for the caregivers and leading to earlier institutionalization [3,4,5]. Though cognitive deterioration is a vital aspect of PSP and CBS, the mechanism of this process is not adequately explored. The issue was brought up in a work highlighting the hypoperfusion of the hippocampus in patients with PSP and CBS affected by glycemic variability [6]. The studies regarding the impact of glycemic variability in other entities indicate its link with medial temporal atrophy [7]. Nevertheless, the mechanism of cognitive deterioration, though possibly related to other tauopathies, should not be interpreted as fully resembling them due to structural discrepancies in histopathological examination. This leads to the necessity to search for tools enabling one to evaluate the course of this process in PSP and CBS patients.

Atypical Parkinsonian syndromes including PSP and CBS currently remain incurable with only symptomatic treatment available. Cognitive symptoms in those entities typically include impaired abstract reasoning and verbal fluency in PSP and ideomotor apraxia with attentional/visuospatial and language deficits in CBS [8]. Pharmacotherapy recommended for cognitive impairment in PSP/CBS is distinct from the one used in other dementias. Commonly prescribed cholinesterase inhibitors could only be used in the case of dominant memory loss. However, those drugs may worsen postural stability [8]. Memantine could impair cognitive abilities in PSP and CBS [8], and therefore should not be used. Treatment usually includes selective serotonin reuptake inhibitors (SSRIs); mood stabilizers; and non-pharmacological approaches such as cognitive training, daily routine maintenance, exercise, proper sleep and nutrition and education of patients and caregivers in terms of the management of executive dysfunction.

As both PSP and CBS are progressive neurodegenerative disorders with almost none or short-lasting responses to pharmacotherapy, all actions aimed at preserving cognitive abilities and reducing the dynamics of disease progression should be taken into consideration during the management of atypical Parkinsonian syndrome (APS) patients. Those interventions should include potentially modifiable factors which could negatively impact cognition, i.a., metabolic disorders.

The association between impaired glucose metabolism and the risk of dementia development has already been proven in the literature. Some papers indicate diabetes itself as the main factor increasing the probability of cognitive impairment [9,10]. Multiple papers analyze the correlations between diabetes type 2 and AD, as both diseases share several features, i.e., insulin resistance, increased oxidative stress reactions, mitochondrial dysfunction, neuronal degeneration and cognitive impairment [11]. Patients with AD and diabetes have higher levels of interleukin 6 (IL-6) in cerebrospinal fluid (CSF), more pronounced tau hyperphosphorylation, increased accumulation of beta-amyloid and more ischemic lesions in the central nervous system (CNS) in comparison with patients with AD and normal glucose metabolism [11]. Diabetes increases the risk of dementia due to vascular and non-vascular mechanisms, such as hypo- and hyperglycemia and central insulin resistance (sometimes called brain diabetes). Multiple studies conducted on animals indicated that insulin administration causes memory improvement; however, it is necessary to maintain normoglycemia [11]. Insulin receptors located in the CNS do not regulate glucose uptake as glucose transporters (GLUT-1 and GLUT-3) on the CNS cells are insulin-independent. CNS insulin receptors modulate synaptic connectivity by having an impact on GABA- and glutamate-mediated transmission [11]. Insulin has neuroprotective effects in the context of neurodegenerative diseases, as it inhibits beta-amyloid secretion [12] and reduces the accumulation of hyperphosphorylated tau [13]. Animal studies indicated the important role of dopamine in cognitive function, as a reduction in dopaminergic transmission causes deterioration in spatial-memory tasks and working memory impairment [14]. Glucose homeostasis is connected with the brain’s monoaminergic system including dopamine metabolism [14], as insulin protects dopaminergic neurons from oxidative stress or mitochondrial dysfunction and participates in dopamine synthesis and turnover [14]. On the one hand, chronic hyperglycemia induces apoptosis of dopaminergic neurons due to oxidative damage. On the other hand, hypoglycemia reduces dopamine release [14]. Therefore, dopaminergic functions are altered in patients with an impaired glucose metabolism, which could be even more pronounced among patients with primary dopamine impairment, as observed in Parkinsonian syndromes. Poor glycemic control in long-term observation leads to cognitive decline and impacts many neurotransmitters, including the dopaminergic system. The contemporary literature predominantly focuses on the impact of the impaired glucose metabolism on cognition itself or describes possible correlations with AD. Less is known in the context of atypical Parkinsonian syndromes. Growing interest is associated with the significance of C-peptide in neurodegenerative disorders. C-peptide is a parameter indicating endogenous insulin secretion [15]. It highlights the functioning of pancreatic beta cells. The role of the C-peptide in neurodegeneration is not recognized. Research assessing the insulin-linked metabolic pathways in AD revealed the inhibitory effect of Braak stage severity of AD on C-peptide brain expression [16]. The role of the C-peptide in tauopathic Parkinsonian syndromes was not verified. Nevertheless, the possible links of the patomechanisms of AD and tauopathic Parkinsonian syndromes may suggest that glycemic variability and abnormalities in the level of C-peptide could be factors playing a role in enhancing the pace of clinical deterioration in the course of the neurodegenerative diseases.

The aim of this study is to evaluate the association between impaired glucose metabolism and cognitive decline among patients with PSP and CBS. The authors of the study hypothesize that increased glucose variability would have a negative impact on cognitive performance among patients with PSP and CBS.

## 2. Materials and Methods

The research was designed as a cross-sectional single-center study. All patients were recruited during the COVID-19 pandemic in Poland, that is between 2022 and 2023. The study included 22 patients: 6 with CBS, 8 with PSP-Parkinsonism predominant (PSP-P) and 8 with PSP—Richardson’s syndrome (PSP-RS). All study participants were hospitalized in the Department of Neurology, Medical University of Warsaw. All diagnoses were based on current criteria [1,2] and none were confirmed by neuropathological examination as all patients remain alive. The analyzed population consisted of 11 females and 11 males with disease duration varying from 3 to 6 years. All patients had completed from 12 up to 15 years of education. Patients with a previous diagnosis of diabetes or other factors with a possible impact on cognitive abilities (e.g., history of neuroinfection, vitamin B deficiency, stroke, brain injury, neurosurgical procedures) were excluded from the study. None of the patients included in the study were active smokers. Only 3 study participants used to smoke cigarettes in the past; however, the duration ranged from 2 to 4 years with more than 10 years without smoking at the time of recruitment. Seven patients were treated for hypertension. However, in all cases, it started >5 years before the onset of the first symptoms of neurodegenerative disease. Moreover, blood pressure was well-controlled with values <140/90 mmHg without any drug modification for the last 3 years. For all study participants, the lipid profile was normal or very slightly increased with lipid concentrations not exceeding the standards by more than 10%. No other cardiovascular risk factors were noted; therefore, the authors decided not to include the above-mentioned parameters in the analysis. Characteristics of the analyzed population are presented in Table 1, in Section 3.

Blood and urine samples were collected and analyzed in the Department of Laboratory Diagnostics of the Mazovian Brodno Hospital in Warsaw. Among all patients, the levels of glycated hemoglobin (HbA1C), fasting blood glucose and fasting C-peptide were evaluated. Urine samples were tested for microalbuminuria. All study participants underwent an oral glucose tolerance test (OGTT) with 75 g of anhydrous glucose in 250 mL of water. Based on the OGTT results, glycemic variability defined as the difference between the highest and lowest level of measured glucose, mean glycemia, glycemia standard deviation (SD) and the coefficient of variation (%CV) defined as ratio of glycemia SD to mean glycemia multiplied by 100%, were calculated. All patients underwent a neuropsychological cognitive assessment with the use of standardized scales—Montreal Cognitive Assessment (MoCA), Mini-Mental State Examination (MMSE) and Frontal Assessment Battery (FAB).

The MoCA scale was created as a brief screening test for mild cognitive impairment (MCI) [17]. The MoCA is a paper-and-pencil test which can be performed in about 10 min. The scale consists of tasks assessing the following cognitive domains: short-term memory, visuospatial abilities, phonemic fluency, verbal reasoning, attention, working memory, naming and orientation in time and place. The total possible score to obtain in the MoCA is 30 points. A score below 26 points suggests deficits in cognitive functioning [18].

The MoCA is a validated tool that has been demonstrated to be efficacious in detecting early and subtle cognitive alterations and can be used as a screening method in both PD and atypical Parkinsonian syndromes [19].

The Mini-Mental State Examination (MMSE) is the most commonly used screening test assessing cognitive functioning in different groups of patients [20]. It is brief and easy to administer. The MMSE has good reliability, but its validity may be weak in some samples like PD [21].

The Frontal Assessment Battery (FAB) has been developed as a brief test of executive function that can be administered at the bedside [22].

The FAB consists of six subtests: verbal conceptualization, verbal fluency, motor programming, sensitivity to interference, inhibitory control and environmental autonomy. Each of the subtests is able to examine a specific cognitive or behavioral domain related to the frontal lobes. A low score (usually below 12) indicates executive dysfunction.

The research shows that the FAB score correlates significantly with other neuropsychological measures of executive functioning, such as the Wisconsin Card Sorting Test and the phonemic and semantic verbal fluency test [23].

The FAB is broadly used as a tool for the assessment of executive function and may provide useful information for differential diagnoses in several diseases which affect the frontal lobes [24], i.a., PSP [25].

The choice of psychological tests was dictated by the desire to assess the usefulness of simple assessment methods available in outpatient settings.

Additionally, the FAB and MoCA scales were divided by an experienced neuropsychologist into subitems evaluating specific areas of cognition. The FAB was divided into similarities, motor series programming and inhibition (sensitivity to interference, inhibitory control, environmental autonomy). The MoCA was divided into orientation, language (naming, language repetition), memory (delayed recall), attention, verbal fluency, abstraction (similarities) and visuospatial functions.

All study participants underwent a Magnetic Resonance (MRI) examination of the brain using the 3T Siemens system in the Department of Radiology at Mazowiecki Szpital Brodnowski. MRI scans were evaluated by a radiologist with 5 years of experience in neuroradiology. As the standard protocol of the brain examination in the Department does not include a T1-weighted sequence in the coronal plane, the atrophy of the medial temporal lobe was assessed according to the Medial Temporal Atrophy Visual Rating Scale [26] in the T2-weighted sequence in coronal plane at the level of the anterior part of the pons and categorized into five stages: 0—no visual atrophy, 1—widening of the choroid fissure, 2—widening of the choroid fissure and temporal horn of the lateral ventricle, 3—moderate atrophy of the hippocampus, 4—severe atrophy of the hippocampus. Additionally, MRI scans were evaluated in order to assess the severity of cerebrovascular lesions with the use of Fazekas scale [27] and categorized into four grades: 0—vascular lesions absent, 1—punctate lesions, 2—beginning confluence, 3—large confluent areas.

The obtained data were analyzed statistically. All statistical analyses and plots were generated using the GraphPad Prism software (version 8.4.3, GraphPad Software, Boston, MA, USA). A comparison between the two tested groups was performed using the Mann–Whitney test. A Mann–Whitney U test was used to assess the differences between demographic and clinical data. For more than two groups, quantitative variables were compared using the Kruskal–Wallis test and Dunn’s post hoc test. Correlations were illustrated using Pearson’s coefficient. To understand the relationship between the MMSE, FAB, MoCA score and a combination of tested biomarkers and other demographic variables, we used multiple linear regression. The results were expressed as the mean ± SD and considered statistically significant at *p* < 0.05.

Due to the rarity of analyzed entities and similarities in their clinical courses, for statistical purposes, the study population was not divided into subgroups based on final diagnosis.

The study was conducted in accordance with the local legislation and institutional requirements. The participants provided written informed consent to participate in this study. The study was performed in accordance with the Declaration of Helsinki and was approved by the Bioethical Committee of the Medical University of Warsaw, KB/109/2022.

## 3. Results

### 3.1. Characteristics of the Analyzed Population

Basic demographical data describing characteristics of the analyzed population are presented in Table 1.

**Table 1:** Characteristics of the analyzed population.

	PSP-P	PSP-RS	CBS
Number of participants	8	8	6
Age [years] (mean)	55–80 (66.5)	60–76 (65.75)	74–80 (70.5)
Sex (male:female)	6:2	3:5	2:4
Disease duration [years] (mean)	3–6 (4.63)	3–6 (3.75)	3–6 (4.5)
Years of education [years] (mean)	12–15 (14)	12–15 (13)	12–15 (14)

### 3.2. Biochemical Assessment

Among the analyzed population, the levels of HbA1C varied from 5.2 to 6.6 with laboratory norm < 6.0. The mean value of HbA1C was 5.6. The pathologically increased value of HbA1C was observed among five study participants.

The fasting glucose level varied from 55 mg% to 151 mg% with a mean value of 97.5 mg%. Among six patients, the fasting glucose level was higher than 100 mg%.

The fasting C-peptide level varied from 0.13 to 5.79 ng/mL with laboratory norm 0.8–4.2 ng/mL. The mean value was 2.4 ng/mL. For two study participants, the fasting C-peptide level was abnormal.

Microalbuminuria defined as a urine microalbumin level above 20 ug/mL was detected among five patients.

### 3.3. OGTT and Parameters Reflecting Glucose Variability

Glycemia 1 h after glucose ingestion varied from 71 to 271 mg% with a mean value of 159.1 mg%. Glycemia 2 h after glucose intake varied from 81 to 261 mg% with a mean value of 162 mg%. Among the analyzed population, 13 patients had hyperglycemia: six patients fulfilled the criteria for impaired glucose tolerance (glucose level within range of 140–199 mg%) and seven fulfilled the criteria for diabetes (glucose ≥ 200 mg%).

Glycemic variability ranged from 22 to 154 mg% with a mean value of 94.7 mg%. Mean glycemia varied from 81.7 to 142 mg% with a mean value of 139.5 mg%.

Glycemia SD ranged from 11 to 88.3 mg% with a mean value of 50.8 mg%.

The coefficient of variation ranged from 13% to 62% with a 40% mean value.

### 3.4. Assessment of Cognition and MRI Results

The neuropsychological assessment with the MoCA varied from 0 to 25 points with a mean value of 16.8 points. All patients had abnormal results.

The results of the MMSE ranged from 0 to 29 points, and the mean result was 23.6 points. The obtained results indicated normal cognition for seven patients, the results of eight patients were interpreted as mild cognitive impairment (24–26 points) and seven patients scored 23 points or less, which indicated dementia.

The FAB results ranged from 0 to 16 points with a mean result of 10 points. All patients scored below the norms adjusted to age and level of education.

The MTA score ranged from 0 to 3 with a mean value of 1.4. The Fazekas score ranged from 0 to 3 with a mean value of 1.

A table summarizing the obtained results was added in Supplementary Materials (neuropsychological subitems contain raw data).

### 3.5. Statistical Correlations

**Figure 1:**
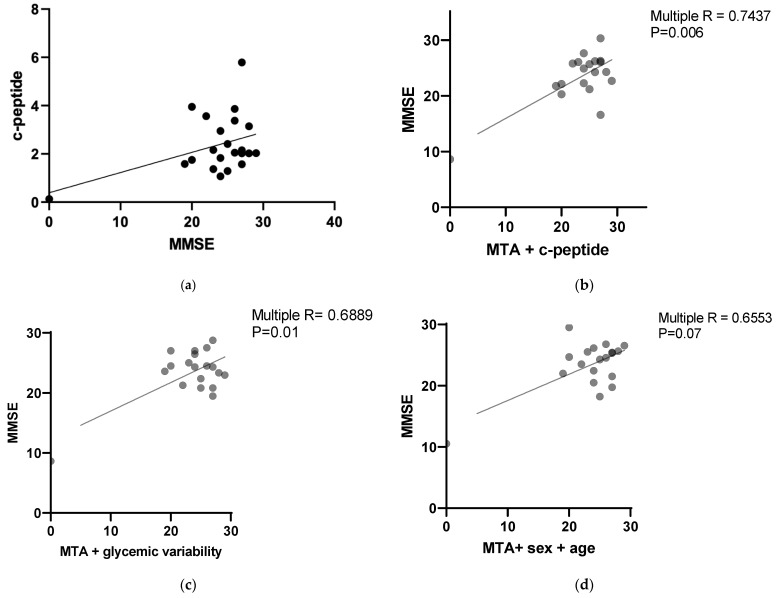

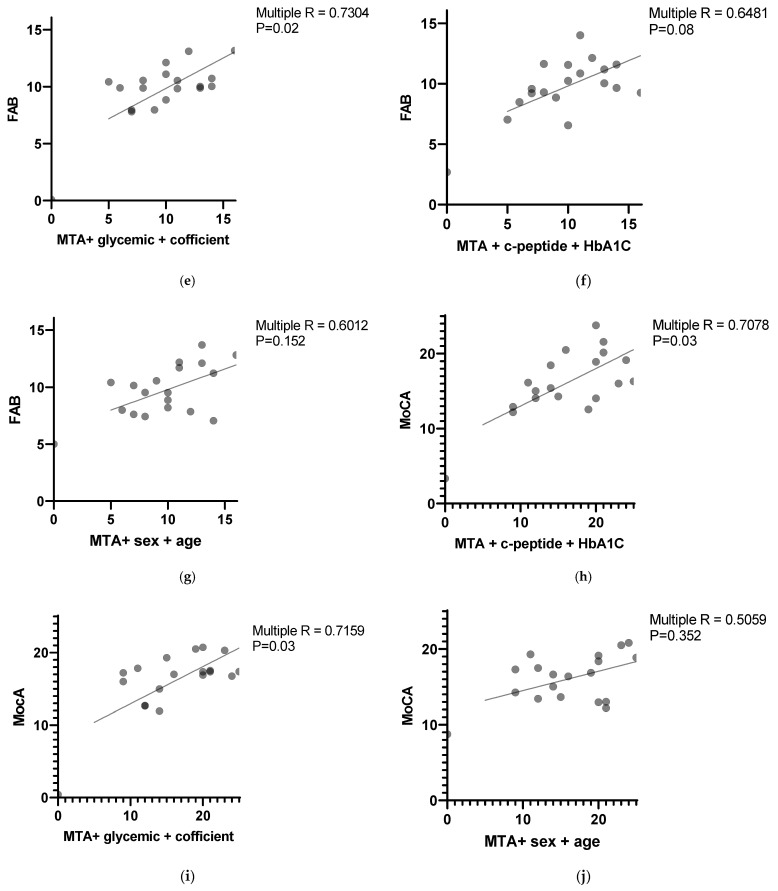
(**a**) Correlation between MMSE results and c-peptide level. *p* = 0.0296 (Pearson r = 0.408312). (**b**) Multiple correlation of MMSE results and MTA score combined with c-peptide level. (**c**) Multiple correlation of MMSE results and MTA score combined with glycemic variability. (**d**) Multiple correlation of MMSE results and MTA score combined with age and sex. (**e**) Multiple correlation of FAB results and MTA score combined with glycemic variability and coefficient of variation. (**f**) Multiple correlation of FAB results and MTA score combined with c-peptide level and HbA1C level. (**g**) Multiple correlation of FAB results and MTA score combined with age and sex. (**h**) Multiple correlation of MoCA results and MTA score combined with c-peptide and Hb1C levels. (**i**) Multiple correlation of MoCA results and MTA score combined with glycemic variability and coefficient of variation. (**j**) Multiple correlation of MoCA results and MTA score combined with age and sex. Each circle represents each patient analyzed.

A statistical analysis revealed a positive correlation between the MMSE results and C-peptide levels with a *p* value 0.0296 (Pearson r = 0.408312). The results are presented in Figure 1a.

Multiple correlation analyses were performed to assess the possible relationships between parameters evaluating cognition and glucose metabolism.

The MMSE results correlated with the MTA score combined with the C-peptide level with a *p* value 0.0060 (multiple R = 0.7437). The results are presented in Figure 1b. The correlation was negative for the MTA score and positive for the C-peptide level.

The MMSE results correlated negatively with the MTA score combined with glycemic variability with a *p* value 0.019 (multiple R = 0.6889). The results are presented in Figure 1c.

No statistically significant correlations were found between the MMSA and MTA scores combined with age and sex (*p* value = 0.0789, multiple R = 0.6553). The results are presented in Figure 1d.

The FAB results correlated negatively with the MTA score combined with glycemic variability and coefficient of variation with a *p* value < 0.022 (multiple R = 0.7304). The results are presented in Figure 1e.

The FAB results correlated with the MTA score combined with the C-peptide level and HbA1C level with *p* value = 0.08 (multiple R = 0.6481). The results are presented in Figure 1f. The correlation was negative for the MTA score and positive for the C-peptide level and HbA1C level.

No statistically significant correlations were found between the FAB and MTA scores combined with age and sex (*p* value = 0.1528, multiple R = 0.6012). The results are presented in Figure 1g.

The MoCA results correlated with the MTA score, C-peptide level and HbA1C with *p* value = 0.0347 (multiple R = 0.7078). The results are presented in Figure 1h. The correlation was negative for the MTA score and positive for the C-peptide level and HbA1c level.

The MoCA results negatively correlated with the MTA score combined with glycemic variability and coefficient of variation with *p* value = <0.03 (multiple R = 0.7159). The results are presented in Figure 1i.

No statistically significant correlations were found between the MoCA and MTA scores combined with age and sex (*p* value = 0.352, multiple R = 0.5059). The results are presented in Figure 1j.

**Figure 2:**
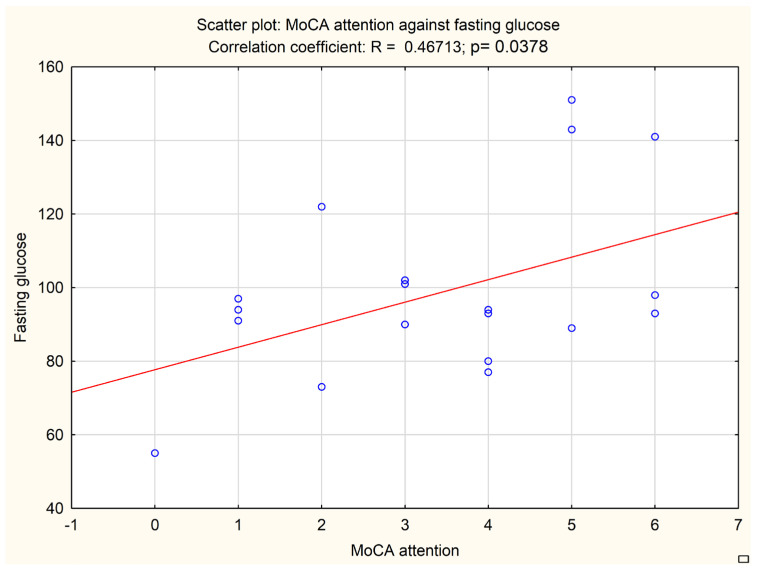
Correlation between MoCA attention subitem and fasting glucose level. Circles represent each patient analyzed, the line represents the correlation.

**Figure 3:**
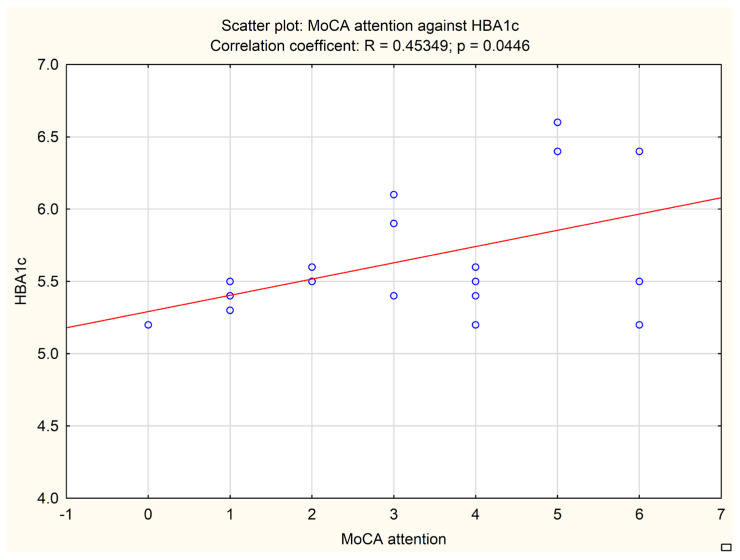
Correlation between MoCA attention subitem and HbA1C level. Circles represent each patient analyzed, the line represents the correlation.

An additional analysis was performed to correlate the subitems of neuropsychological tests with biochemical and imaging data. A MoCA subitem evaluating attention correlated with the fasting glucose level (*p* = 0.0378, Pearson r = 0.46713) and HbA1c level (*p* = 0.0446, Pearson r = 0.45349). The results are presented in Figure 2 and Figure 3.

**Figure 4:**
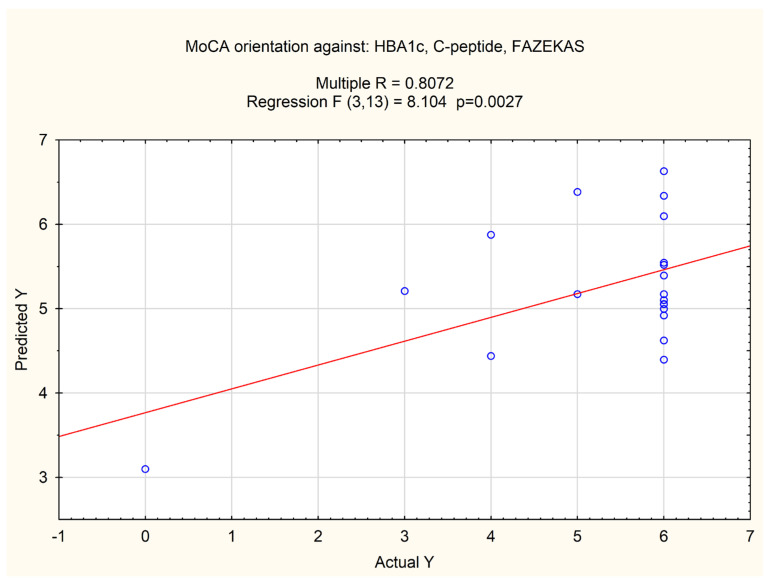
Multiple correlation of MoCA orientation subitem and HbA1C level combined with C-peptide level and Fazekas score. Circles represent each patient analyzed, the line represents the correlation.

A multivariate analysis based on linear regression revealed that the MoCA subitem evaluating orientation correlated with the HbA1C level combined with the C-peptide level and Fazekas score (*p* value = 0.0027, multiple R = 0.8072). The results are presented in Figure 4.

No other correlations were found to be statistically significant.

The outcome of the study could be a result of copathologies related to vascular and neurodegenerative damage.

## 4. Discussion

The obtained results indicate that poor control of glycemia with high glycemic variability and increased atrophy of the medial temporal lobe among patients with PSP and CBS correlate with worse cognitive performance independent of age or sex, even among patients who do not fulfill the criteria for diabetes. Similar results were obtained from the examination of 461 patients from a memory clinic [7]. However, that study [7] did not provide details concerning final diagnoses of analyzed patients, with the exception of dividing the analyzed group into mild cognitive impairment, subjective cognitive impairment, probable AD, probable vascular dementia and untyped dementia. The correlation between glucose variability and future dementia diagnosis was found in a 10-year retrospective study conducted on Latinx adults with diabetes mellitus type 2 (T2DM) [28]. Blood glucose variability was associated with cognitive dysfunction and the level of lacunar infarctions’ burden among patients with T2DM [29]. Therefore, it could be hypothesized that an escalated risk of vascular cerebral complications is at least one of the possible mechanisms connecting increased glucose variability and poor cognitive outcome. T2DM was associated with earlier onset of vascular dementia and faster deterioration of cognition [30]. Diabetes with poor glycemic control was associated with a two-fold increased risk of mild cognitive impairment and a three-fold increased risk of progression to dementia [31]. Among the different cognitive areas assessed by the subitems of neuropsychological tests used in the study, only MoCA orientation and attention parts were found to directly correlate with glycemic and imaging parameters. This could be interpreted as an argument confirming the possible usefulness of the MoCA scale among PSP/CBS patients. However, in order to fully evaluate the impact of glucose metabolism impairment on specific domains of cognitive impairment among patients with tauopathic Parkinsonian syndromes, a more sophisticated neuropsychological assessment would be required.

The worsening of glycemic control described as an HbA1c increase was associated with cognitive decline even among non-diabetic elderly patients [32]. However, this paper does not describe the possible impact of glycemic variability. It has been found that glucose variability is associated with AD development [33]; however, there are no data concerning cognitive impairment due to atypical Parkinsonian syndromes.

The issue concerning the significance of glycemic variability in atypical Parkinsonian syndromes is rarely discussed. The outcome of the study partly comes up with previous results of the assessment of cerebral perfusion among patients with PSP or CBS with or without glycemic variability [6]. In this study, the authors revealed more pronounced hypoperfusion of the hippocampus in patients with increased levels of glycated hemoglobin. Hypoperfusion was interpreted as a state possibly preceding atrophic change. The research was based on a single assessment of the patients. It was limited by a relatively small number of patients and the lack of neuropathological verification. To the best of our knowledge, the issue of tauopathic Parkinsonian syndrome in the context of glycemic variability was not further explored. The significance of glycemic variability in tauopathies is not a widely described issue. A Taiwan diabetes study interpreted it as a risk factor of AD [33]. A different study showed that the link between hyperglycemia and cognitive deterioration cannot be observed among patients under regular health control [34]. On the other hand, prediabetes early intervention may be interpreted as a factor enabling the preservation of hippocampal subfields [35]; therefore, it could be useful in managing patients with primarily neurodegenerative diseases.

The results from the study confirm the positive impact of insulin secretion on cognition, as the level of C-peptide positively correlated with the neuropsychological assessment (MMSE, MoCA, FAB scores). This is in line with the previously described role of insulin in the modulation of synaptic connectivity [11]. Interestingly, the FAB and MoCA scores correlated positively with the level of HbA1c, which could suggest the significance of hypoglycemia rather than hyperglycemia in the context of cognitive deterioration. The prominent impact of hypoglycemic episodes on dementia development among patients with T2DM has already been determined [36,37]. However, this hypothesis requires further research among patients with APS.

The results obtained in this study are similar to observations made on the elderly Chinese population [38]. The study found that patients over 65 years old with T2DM, who underwent intensified glycemic control treatment determined to reduce the level of HbA1C by ≥10%, had higher incidence of dementia in comparison with patients with less-intense treatment goals. However, it has been established that for patients with diabetes, the presence of glucose peaks increases the incidence of developing dementia regardless of the HbA1C level [39]. A meta-analysis including 577,592 patients with diabetes, predominantly type 2, revealed that a higher visit-to-visit variability of HbA1c is associated with an increased risk of developing dementia [40]. A high variability of HbA1c was found to be a risk factor for developing dementia and was correlated with a lower hippocampal volume among middle aged and elders without diabetes [41], which highlights the importance of monitoring glycemic variability independently from the diagnosis of impaired glucose metabolism.

To the best of our knowledge, currently there are no manuscripts available that would directly address the issue concerning the impact of glucose metabolism impairment on the clinical course of atypical Parkinsonian syndromes, especially the pace of cognitive deterioration. Because of the lack of the literature describing presumptive differences in this population, it could be assumed that general mechanisms responsible for cognitive deterioration among patients with poor glycemic control are universal and are present also among patients with PSP and CSB. This indicates the importance of routine repetitive screening focused on the detection and treatment of metabolic abnormalities and a multidisciplinary approach in everyday healthcare dedicated to patients with atypical Parkinsonian syndromes.

The study, although providing some insight on a previously unexplored matter, is limited by several aspects. Firstly, the analyzed population was relatively small; however, this is caused by the rarity of the analyzed diseases. All data were obtained from a single center, and the enrollment was conducted during the COVID-19 pandemic, which limited the number of planned hospital admissions. Due to the restricted funds and limited hospital admissions caused by the COVID-19 pandemic during the recruitment period, the study focused mainly on patients with atypical Parkinsonian syndromes without a healthy control group. The obtained correlations between the analyzed factors were high or moderate, which could be caused by the relatively small number of analyzed patients. However, data presented in the study signpost a direction for future research concerning the impact of metabolic abnormalities on the pace of deterioration in tauopathies. The presented results should be treated as preliminary. However, due to the lack of an effective therapy for PSP and CBS patients and the high accessibility of a glucose metabolism assessment with a relatively unchallenging intervention in the case of metabolic abnormalities, the obtained data could be useful in everyday clinical practice. The authors, aware of this limitation, are planning to conduct a multi-center study in order to confirm their observations with a larger population. Secondly, diagnoses were made based on clinical criteria and were not confirmed by neuropathological examination. All study participants remain alive; therefore, no such examination could be conducted. This lack of neuropathological verification limited the possibility of an analysis of likely coexisting neurodegenerative and vascular changes. This led to difficulties in the evaluation of patomechanisms which resulted in the clinical outcome, which can be observed among patients, of cognitive decline. Though distinguishing the elements related to one of the pathologies was not possible in the in vivo evaluation, the examination of the features interpreted as risk factors of neurodegenerative and vascular diseases seems relevant. Interestingly, among factors increasing the risk of PSP and CBS development, glucose metabolism impairment is mentioned [42]. Another limitation of the study was the lack of continuous glucose monitoring. All parameters calculated for the study were based on blood glucose level measurements made during the OGTT or during a singular assessment of fasting glucose. However, it could be assumed that if continuous glucose monitoring was used, glycemic variability among the analyzed patients would be alike or even higher than that presented in the study.

## 5. Conclusions

The results obtained in the study indicate the possible importance of glucose metabolism control and optimal treatment in the context of cognition maintenance among patients with PSP and CBS. The preliminary data presented in the study should be considered as the signpost indicating a possibly novel approach to the management of neurodegenerative disorders, which would include awareness of the possible impact of metabolic abnormalities on such disorders. To the best of our knowledge, this is the first study discussing the role of glycemic variability in atypical Parkinsonian syndromes, and this issue requires further assessment. However, due to the current lack of causal treatment and low effectiveness of symptomatic therapies in PSP and CBS, a multidisciplinary approach including dietary interventions and the optimalization of metabolic disorders should be considered in the optimific care of patients with atypical Parkinsonian syndromes such as PSP and CBS.

## Data Availability

The datasets generated and/or analyzed during the current study are available from the corresponding author on reasonable request.

## Supplementary Materials

The following supporting information can be downloaded at: https://www.mdpi.com/article/10.3390/jcm13020465/s1. Table S1: Results obtained—raw data.
